# A rare case of endogenous *Aspergillus conicus* endophthalmitis in an immunocompromised patient

**DOI:** 10.1186/1869-5760-3-37

**Published:** 2013-02-11

**Authors:** Wendy M Smith, Gary Fahle, Robert B Nussenblatt, Hatice Nida Sen

**Affiliations:** 1 Laboratory of Immunology, National Eye Institute, National Institutes of Health, 10 Center Dr, 10/10N112, Bethesda, MD, 20892, USA; 2 Department of Laboratory Medicine, Clinical Center, National Institutes of Health, Bethesda, MD, 20892, USA

**Keywords:** *Aspergillus*, Immunocompromise, Panuveitis, HIV, Polymerase chain reaction

## Abstract

**Background:**

The purpose of this study was to report the case of a patient with bilateral panuveitis who was found to have a rarely reported intraocular fungus, *Aspergillus conicus*. A 40-year-old man presented with gradual vision loss in both eyes. He had bilateral anterior uveitis, granulomatous vitritis with a preretinal granuloma in the right eye, and nongranulomatous vitritis with two quadrants of chorioretinal scarring in the left.

**Findings:**

Serological testing revealed a new diagnosis of human immunodeficiency virus as well as positive rapid plasma reagin and fluorescent treponemal antibody. Polymerase chain reaction (PCR) testing of the aqueous humor from the right eye identified *A. conicus*. Due to the indolent course of the endogenous fungal infection, the patient was treated with prednisolone acetate 1% eye drops, oral voriconazole, and highly active antiretroviral therapy. More than 1 year later, his vision remained 20/20 in both eyes without any episodes of recurrent inflammation.

**Conclusions:**

PCR testing helped identify a rare intraocular infection in an immunocompromised patient. In this case, *A*. *conicus* behaved less aggressively than other species of *Aspergillus* implicated in intraocular infection.

## Findings

### Introduction

*Aspergillus* is the second most common cause of endogenous fungal endophthalmitis after *Candida* and is more likely to occur in immunocompromised patients due to HIV, malignancy, diabetes mellitus, or immunosuppressive medications [1]. Other associations include intravenous drug use, solid organ transplantation, lung disease, and renal insufficiency; however, *Aspergillus* endophthalmitis may occur in immunocompetent individuals with no known risk factors [2]. In general, the prognosis is poor for preservation of vision and even the eye itself [3]. Disseminated aspergillosis can be fatal, but isolated ophthalmic disease is not uncommon, especially among immunocompetent patients. We describe a patient who presented with bilateral panuveitis and was subsequently diagnosed with HIV. Polymerase chain reaction (PCR) testing of the aqueous humor (AH) identified *Aspergillus conicus*, a species rarely associated with intraocular infection.

### Case report

A 40-year-old white man was referred to our uveitis clinic with several weeks of painless, gradual vision loss in both eyes (OU) and several months of low-grade fevers and weight loss. Five years prior to presentation, he had been diagnosed with uveitis and syphilis (rapid plasma reagin (RPR) 1:1,024). After treatment with prednisolone acetate 1% eye drops and three intramuscular penicillin injections, his vision returned to normal, but he did not return for follow-up. Just prior to referral to our clinic, his vision was 20/50 in the right eye (OD) and 20/60 in the left (OS); prednisolone acetate 1% drops were started OU.

**Figure 1:**
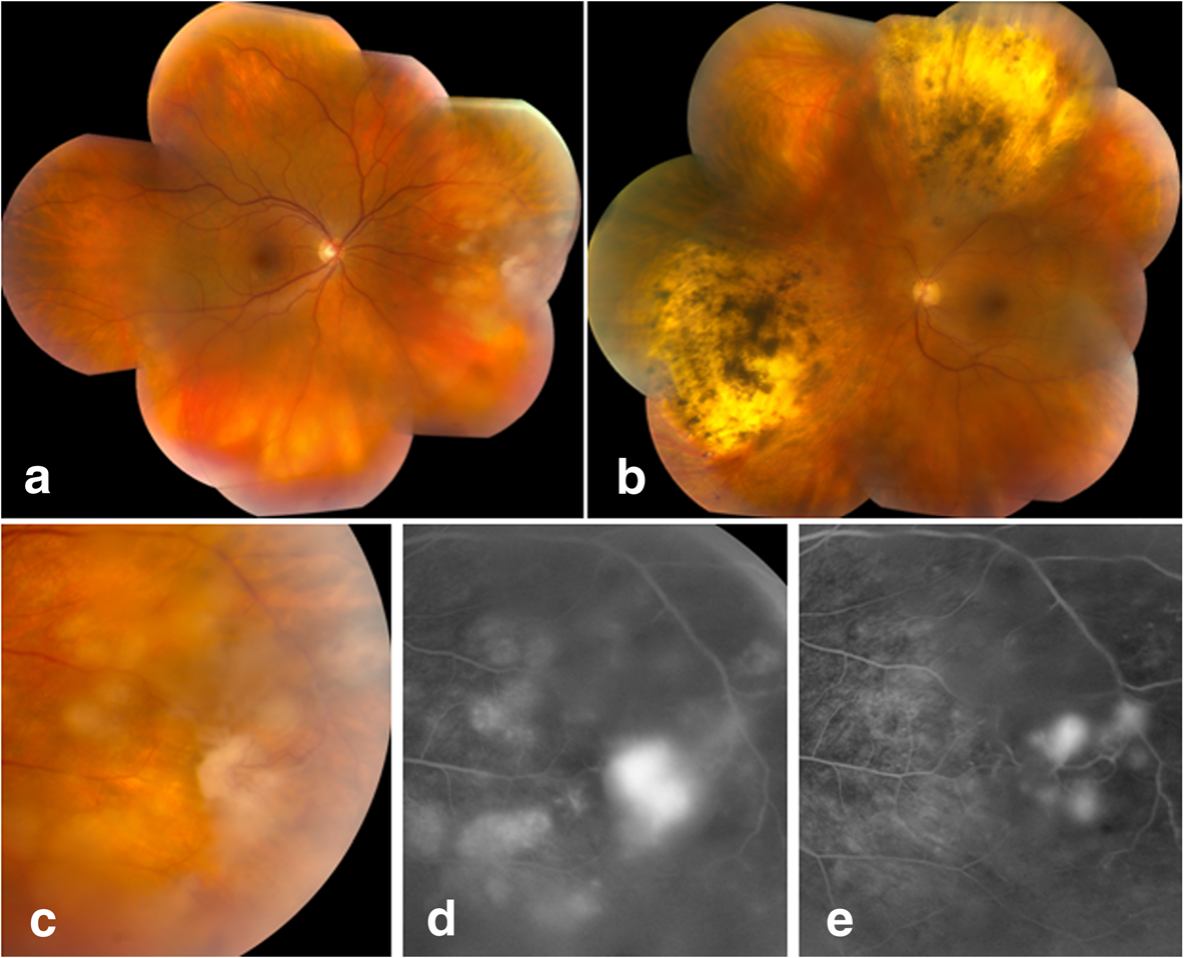
**Color montages upon presentation and FA images upon presentation and after treatment. **Color montages of the right (**a**) and left (**b**) eyes upon presentation to our clinic. Detail of the color (**c**) and FA (**d**) images of the right eye on presentation: The fluffy lesion appeared to emanate from a retinal vessel with reactive, adherent vitreous. FA (**e**) of the right eye 6 weeks after starting HAART, voriconazole, and intravenous penicillin.

On examination, visual acuity was 20/20 OD and 20/32 OS. There were trace cells in both anterior chambers. Both eyes also had 1+ vitreous cell and trace haze (based on the National Eye Institute vitreous grading scale) [4], but the OD vitreous cell had distinct granulomatous-appearing clumps. In the nasal periphery OD, there was a fluffy, white, preretinal lesion with several adjacent creamy, white, deep retinal lesions and intraretinal hemorrhages (Figure 1a,c). The superior and inferior nasal retina OS had large, wedge-shaped quadrants of pigmented chorioretinal atrophy and scarring (Figure 1b). Fluorescein angiography (FA) showed hyperfluorescence of the preretinal granuloma and the surrounding deep retinal lesions OD (Figure 1d) and staining of the chorioretinal scars, focal vasculitis, and minimal perifoveal leakage OS.

On physical examination, he had severe fingernail onychomycosis, oral thrush, and multiple raised, red cutaneous lesions on his extremities and trunk. Further history revealed a chronic cough for several years; he quit smoking methamphetamines 6 years earlier. He also had a history of same-sex partners. Lab testing revealed a reactive HIV1/2 antibody (confirmed by Western blot) and a CD4 count of 158 cells/μL. RPR was reactive (1:2) with a positive syphilis IgG and reactive FTA-ABS. The following were negative: QuantiFERON-TB, *Toxoplasma* IgG, and *Toxocara* titer. PCR of AH OD was negative for CMV, VZV, HSV, and toxoplasma and positive for a panfungal amplicon using the internal transcribed spacer, a 500-bp sequence, and a highly variable region of the fungal ribosomal DNA as the primers. Sequencing identified *A. conicus*. There was no growth in fungal cultures of AH and peripheral blood.

A chest CT showed diffuse bilateral interstitial opacities and nodular densities, and consolidation in the dependent lower lobes. A bronchoalveolar lavage specimen was negative for the Fungitell assay, *Aspergillus* antigen, *Pneumocystis*, acid-fast stain, and mycobacterial and *Nocardia* cultures. Biopsy of a skin lesion was consistent with Kaposi's sarcoma. A brain MRI was unremarkable.

Since the patient's vision and clinical exam remained stable, prednisolone acetate 1% eye drops were continued in conjunction with continuous intravenous penicillin (2-week course), oral voriconazole 200 mg twice daily, and highly active antiretroviral therapy (HAART). Seven weeks later, the lesion in the right eye appeared less active (Figure 1e). By 4 months, the panuveitis was quiescent with visual acuity 20/20 OU. The presumed diagnoses were endogenous *A. conicus* endophthalmitis OD and panuveitis OU possibly due to syphilis (reinfection or insufficient treatment in 2005). The chorioretinal scars OS remained inactive and were likely related to the previous syphilis-related uveitis in 2005. He remained quiet without recurrent uveitis throughout a 1-year course of voriconazole as well as 2 months after stopping the antifungal medication.

The patient signed a research protocol consent (approved by the NIH IRB in accordance with the Helsinki Declaration) which states that data collected from exam visits might be published.

### Discussion and conclusion

*Aspergillus* has a ubiquitous presence in the environment as a saprophytic mold isolated from soil and decaying organic matter [5]. *A*. *fumigatu*s and *A*. *flavus* are the most common species implicated in intraocular infection. Other species detected in ophthalmic disease include *A*. *niger*, *A*. *terreus*, *A*. *ustus*, *A*. *glaucus*, and *A*. *versicolor*[5-8]. The species in our case, *A*. *conicus*, is rarely mentioned in the medical literature, and the only previous report of ophthalmic involvement was from a patient with postsurgical endophthalmitis [9]. *A*. *conicus* has also been isolated from the respiratory tracts of healthy people in Japan, Taiwan, and South Korea [10]. Interestingly, our patient did have a history of travel to Korea and Japan where he might have come into contact with *A*. *conicus*.

Treatment for *Aspergillus* endophthalmitis classically involves intravitreal and/or systemic amphotericin B, although the intraocular form has been associated with retinal toxicity [11]. Intravitreal dexamethasone is sometimes administered in conjunction with the intravitreal antifungal medication to reduce intraocular inflammation although the efficacy has not been tested in controlled, masked studies [1,5,12,13]. Pars plana vitrectomy is often performed for diagnostic and therapeutic purposes, especially when the diagnosis is unknown or the vision is severely compromised. Several species can be highly resistant to amphotericin B, and side effects often limit its systemic use. Alternative systemic antifungals including caspofungin and voriconazole can be effective as well, although vitrectomy may still be necessary with extensive, sight-threatening intraocular involvement. Voriconazole has good intraocular penetration allowing systemic treatment alone in selected cases such as ours; when used intravitreally, it may be safer than amphotericin B [11].

In addition to the identification of a rare *Aspergillus* species, *A*. *conicus*, our case differs from others in terms of presentation and outcome. Our patient had gradual, painless vision loss without conjunctival hyperemia or severe anterior chamber inflammation. The disease course was indolent, and systemic treatment alone resulted in good visual acuity that remained stable without recurrent inflammation more than 1 year later. In most cases, *Aspergillus* endophthalmitis requires systemic and intravitreal antifungal treatment as well as vitrectomy, and the visual outcome is poor; however, our patient had nonmacular threatening disease and did not progress on systemic therapy, so surgery and intravitreal antifungals were unnecessary. No other focus of *A*. *conicus* infection was detected, although the lungs were the most likely entry point. It is possible that *A*. *conicus* is not as aggressive as other species implicated in *Aspergillus* endophthalmitis, although the very small number of reported infections does not allow a definitive conclusion. We do not routinely perform repetitive AH sampling on an eye with very good visual acuity if there is an appropriate response to treatment and quiescent inflammation; therefore, we cannot provide direct proof of treatment success in this case.

PCR played an important role in the identification of the presumed causative organism in this case and allowed targeted antifungal therapy rather than just antibiotics for ocular syphilis and HAART for HIV. As the yield from ocular fluid cultures is frequently poor, it is possible that *A*. *conicus* has been responsible for endophthalmitis in cases in which no organism was cultured or the species could not be identified. Many studies have demonstrated the utility of PCR especially since multiplex testing can be performed on a very small volume of fluid [14,15]. Due to the sterile intraocular environment, ocular fluids can be ideal for PCR examination, although environmental contamination by commensal microorganisms is still a potential confounding factor.

In summary, we describe a patient with bilateral panuveitis who was diagnosed with an unusual fungal endophthalmitis and an underlying immunodeficiency due to HIV. Our case emphasizes the importance of a detailed review of systems which may reveal related infectious or inflammatory issues with potentially serious systemic consequences.

## Competing interests

The authors declare that they have no competing interests.

## Authors’ contributions

WMS designed and conducted the study and wrote the manuscript. GF carried out the molecular genetic tests and participated in editing the manuscript. RBN participated in the design of the study and preparation of the manuscript. HNS designed the study and assisted in writing the manuscript. All authors read and approved the final manuscript.
